# Supplementary material for: Phenolic Profile of Herbal Infusion and Polyphenol-Rich Extract from Leaves of the Medicinal Plant Antirhea borbonica: Toxicity Assay Determination in Zebrafish Embryos and Larvae
Source: Molecules. 2020 Sep 29;25(19):4482. doi: 10.3390/molecules25194482 (PMC7583820; doi:10.3390/molecules25194482)
Supplement: Supplementary file 1 [file molecules-25-04482-s001.pdf]

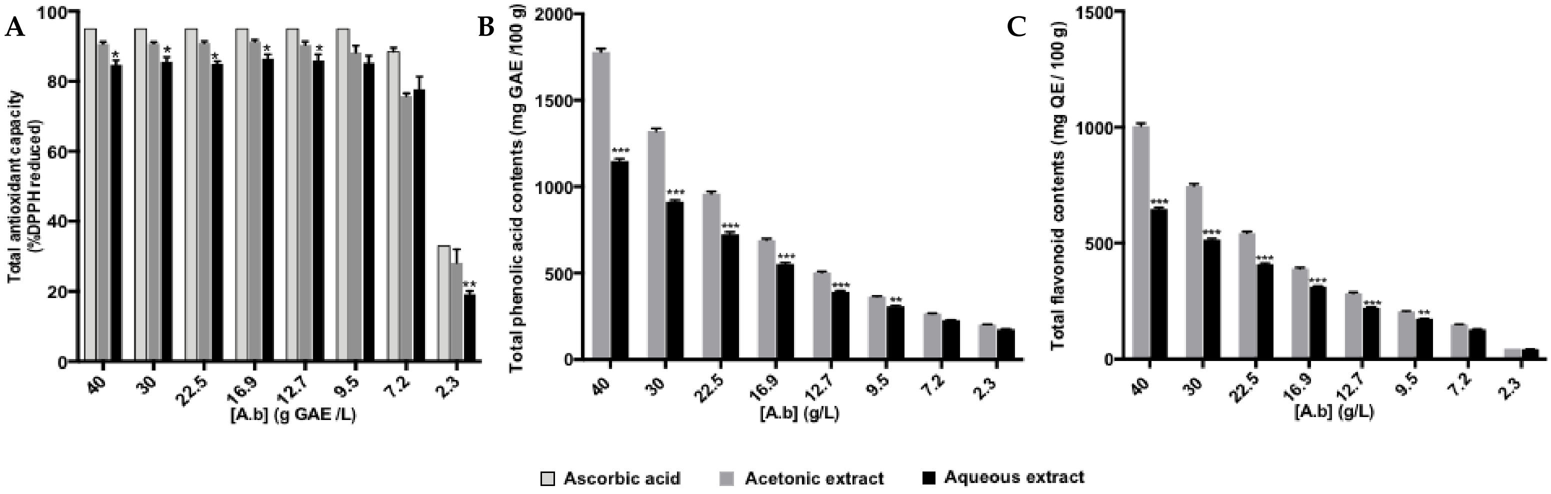

Figure S1. Total phenolic acid, flavonoid content and antioxidant capacity from *A. borbonica* extracts.

(**A**) The total antioxidant capacity of polyphenols-rich extracts from *A. borbonica* (*A.b*) were measured by DPPH assay at different concentrations ranging from 40 to 2.3 g GAE/L. Ascorbic acid was used as positive control. The results were expressed as % DPPH reduced. **(B)** The total phenolic contents of an acetonic and aqueous extracts from *A. borbonica* were determined by using the Folin-Ciocalteu colorimetric assays at different concentrations ranging from 40 to 2.3 g/L (dried plant powder). The results were expressed as mg gallic acid equivalent (GAE)/100 g dried plant powder. **(C)** The total flavonoid contents were determined by using the aluminum chloride colorimetric assay. The results were expressed as mg quercetin equivalent (QE)/100g dried plant powder. Data are mean ± SD of three independent experiments. * $p < 0.05$, ** $p < 0.01$, *** $p < 0.001$ (*vs.* acetonic extract).

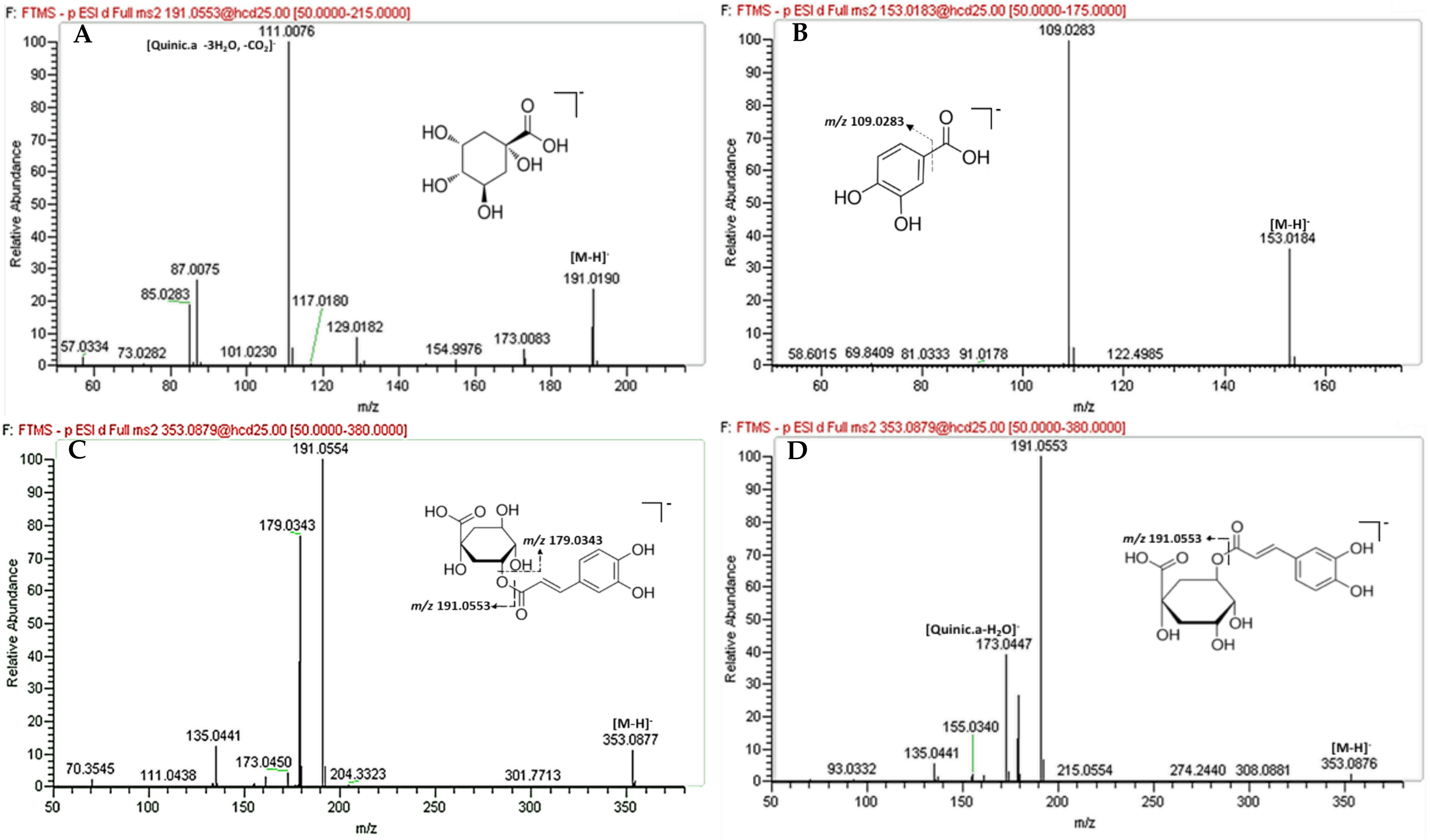
F: FTMS - p ESI d Full ms2 191.0553@hcd25.00 [50.0000-215.0000]
A
[Quinic.a -3H2O, -CO2]-
111.0076
87.0075
85.0283
57.0334
73.0282
101.0230
117.0180
129.0182
154.9976
173.0083
[M-H]-
191.0190
Relative Abundance
m/z
F: FTMS - p ESI d Full ms2 153.0183@hcd25.00 [50.0000-175.0000]
B
109.0283
m/z 109.0283
[M-H]-
153.0184
58.6015
69.8409
81.0333
91.0178
122.4985
F: FTMS - p ESI d Full ms2 353.0879@hcd25.00 [50.0000-380.0000]
C
191.0554
179.0343
m/z 179.0343
m/z 191.0553
135.0441
173.0450
70.3545
111.0438
204.3323
301.7713
[M-H]-
353.0877
F: FTMS - p ESI d Full ms2 353.0879@hcd25.00 [50.0000-380.0000]
D
191.0553
m/z 191.0553
[Quinic.a-H2O]-
173.0447
155.0340
135.0441
93.0332
215.0554
274.2440
308.0881
[M-H]-
353.0876

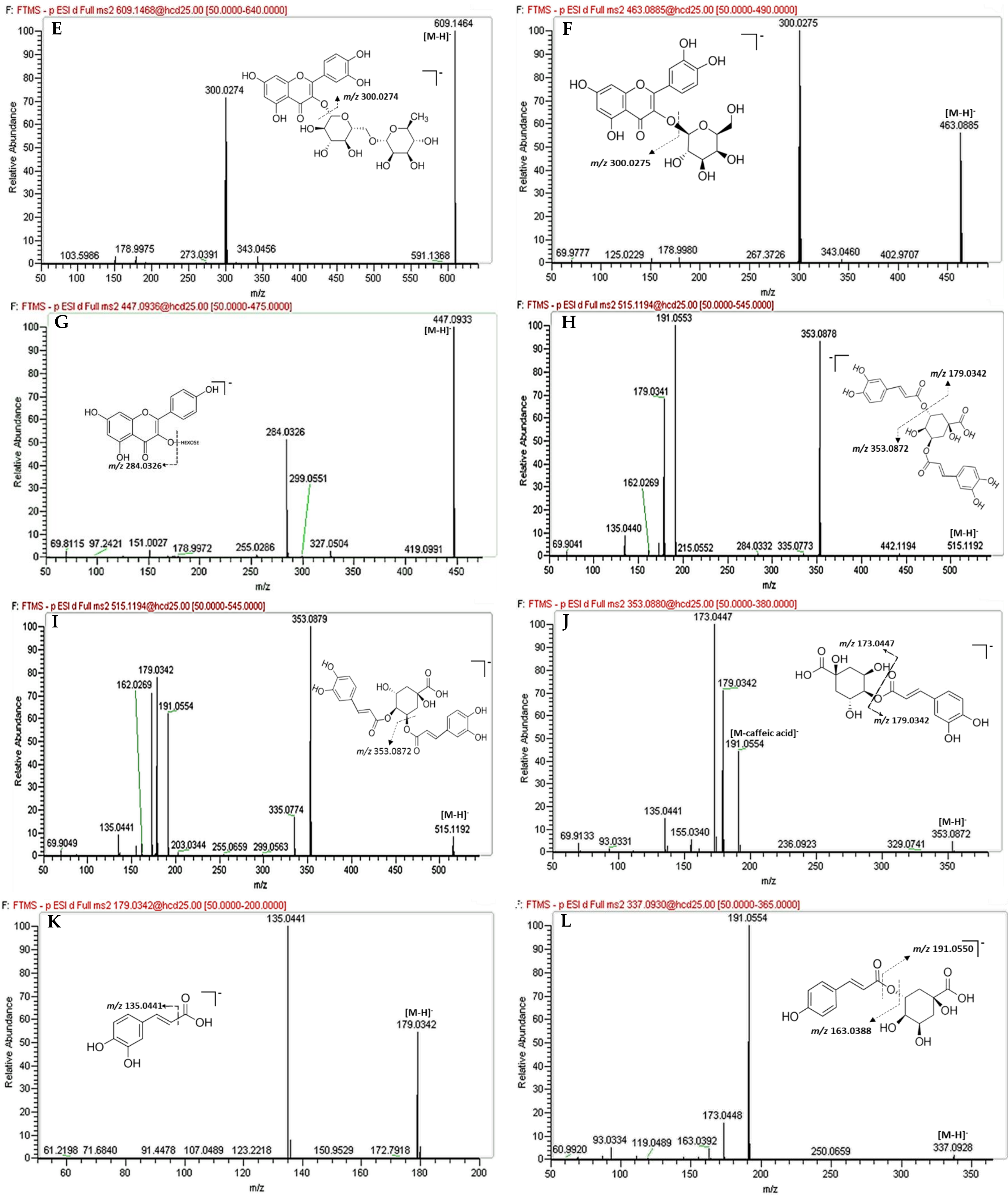
F: FTMS - p ESI d Full ms2 609.1468@hcd25.00 [50.0000-640.0000]
E
609.1464
[M-H]-
300.0274
m/z 300.0274
103.5986
178.9975
273.0391
343.0456
591.1368
F: FTMS - p ESI d Full ms2 463.0885@hcd25.00 [50.0000-490.0000]
F
300.0275
[M-H]-
463.0885
m/z 300.0275
69.9777
125.0229
178.9980
267.3726
343.0460
402.9707
F: FTMS - p ESI d Full ms2 447.0936@hcd25.00 [50.0000-475.0000]
G
447.0933
[M-H]-
284.0326
299.0551
m/z 284.0326
HEXOSE
69.8115
97.2421
151.0027
178.9972
255.0286
327.0504
419.0991
F: FTMS - p ESI d Full ms2 515.1194@hcd25.00 [50.0000-545.0000]
H
191.0553
353.0878
179.0341
162.0269
135.0440
m/z 179.0342
m/z 353.0872
69.9041
215.0552
284.0332
335.0773
442.1194
[M-H]-
515.1192
F: FTMS - p ESI d Full ms2 515.1194@hcd25.00 [50.0000-545.0000]
I
353.0879
179.0342
162.0269
191.0554
m/z 353.0872
335.0774
135.0441
69.9049
203.0344
255.0659
299.0563
[M-H]-
515.1192
F: FTMS - p ESI d Full ms2 353.0880@hcd25.00 [50.0000-380.0000]
J
173.0447
179.0342
[M-caffeic acid]-
191.0554
m/z 173.0447
m/z 179.0342
135.0441
155.0340
69.9133
93.0331
236.0923
329.0741
[M-H]-
353.0872
F: FTMS - p ESI d Full ms2 179.0342@hcd25.00 [50.0000-200.0000]
K
135.0441
m/z 135.0441
[M-H]-
179.0342
61.2198
71.6840
91.4478
107.0489
123.2218
150.9529
172.7918
F: FTMS - p ESI d Full ms2 337.0930@hcd25.00 [50.0000-365.0000]
L
191.0554
m/z 191.0550
m/z 163.0388
173.0448
60.9920
93.0334
119.0489
163.0392
250.0659
[M-H]-
337.0928
Relative Abundance
m/z

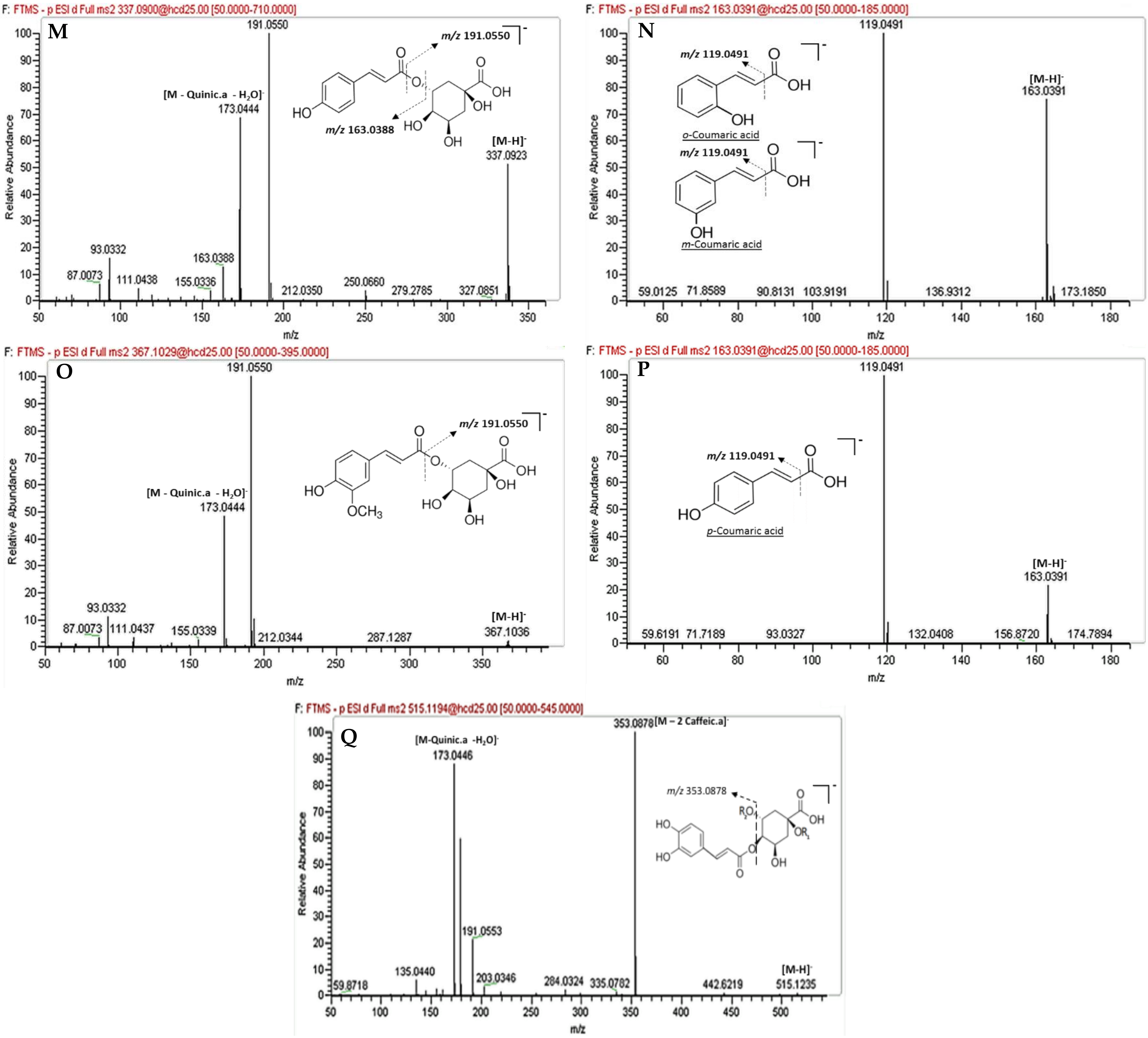

Figure S2. The fragmentation pattern of each identified compounds obtained in negative mode.

(A) Quinic a. **(B)** Protocatechuic a. **(C)** 3-Caffeoylquinic a. **(D)** 5-Caffeoylquinic a. **(E)** Quercetin-3-O-rutinoside **(F)** Quercetin-3-O-glucoside **(G)** Kaempferol-O-hexoside **(H)** 3,5-Dicaffeoylquinic a. **(I)** 3,4-Dicaffeoylquinic a. **(J)** 4-Caffeoylquinic a. **(K)** Caffeic a. **(L)** *p*-Coumaroylquinic a. **(M)** *p*-Coumaroylquinic a. **(N)** *o*/*m*-coumaric a. **(O)** Feruloylquinic a. (-OR_2) **(P)** *p*-coumaric a. **(Q)** 1,4-Dicaffeoylquinic a.(-OR_1) / 4,5-Dicaffeoylquinic.a (-OR_2) .

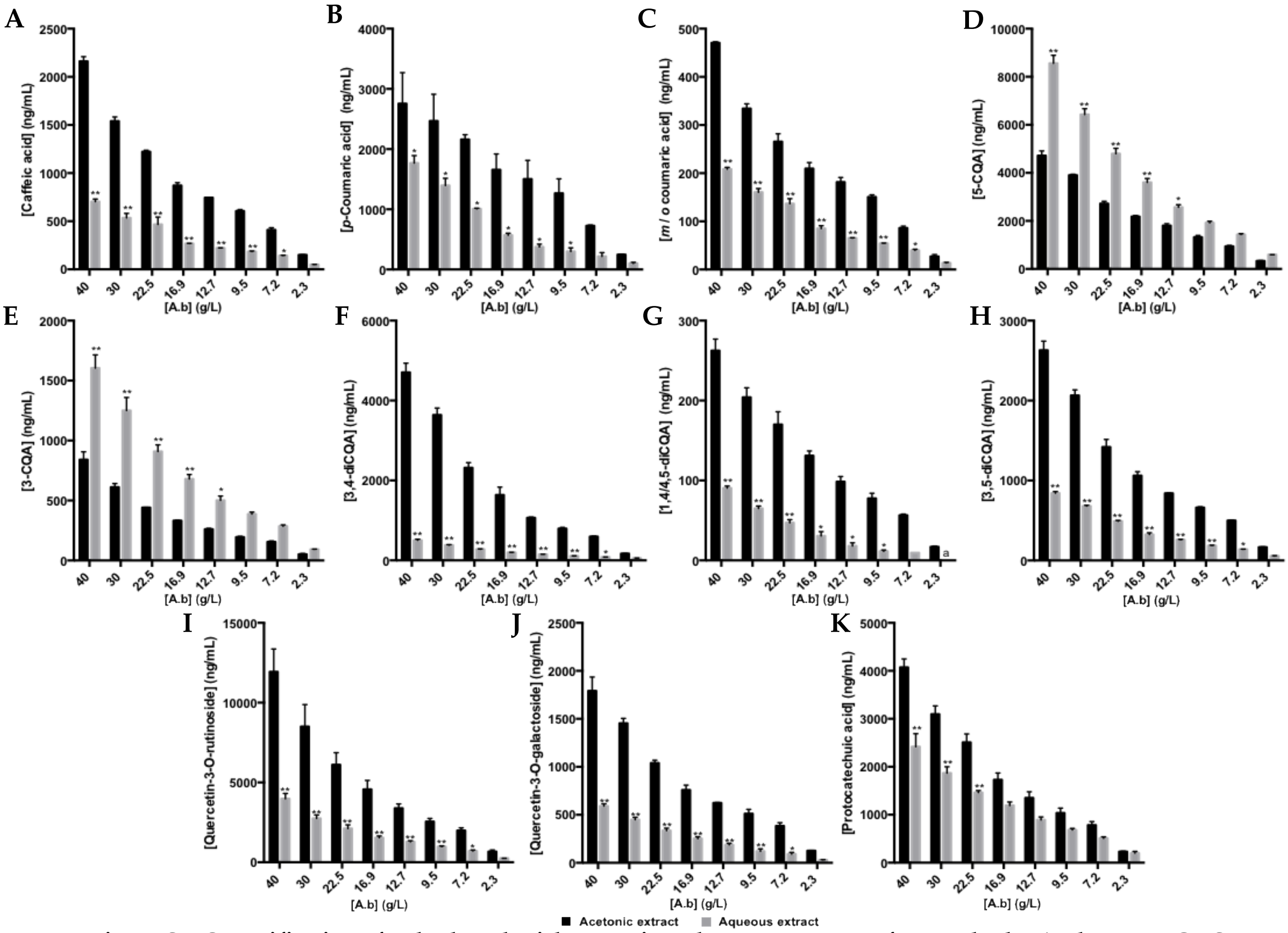

Figure S3. Quantification of polyphenols-rich acetonic and aqueous extracts from *A. borbonica* by UHPLC-ESI-MS.

The analysis were performed by using a Q-Exactive plus mass spectrometer at different concentrations ranging from 40 to 2.3 g/L (dried plant powder). **(A)** Caffeic a. **(B)** *p*-Coumaric a. **(C)** *o*/*m*-Coumaric a. **(D)** 5-Caffeoylquinic a. **(E)** 3-Caffeoylquinic a., **(F)** 3,4-Dicaffeoylquinic a., **(G)** 1,4/4,5-Dicaffeoylquinic a. **(H)** 3,5-Dicaffeoylquinic a. **(I)** Quercetin-3-O-rutinoside **(J)** Quercetin-3-O-glucoside **(K)** Protocatechuic a. The concentrations of compounds were expressed as ng/mL. Data are mean ± SD of three independent experiments. * $p < 0.05$, ** $p < 0.01$ (*vs.* acetonic extract). Item [a]: Under limit of quantification (<LOQ).

Table S1. Identification of 20 compounds in *Antirhea borbonica* herbal infusion by LC-HESI-UV-MS/MS in negative mode

**Compound**	**Molecular formula**	**Mass error (ppm)**	**[M-H]$^-$**
D-(-)-Quinic acid	$C_7H_{12}O_6$	0.4	191.0554
Gallic acid	$C_7H_5O_5$	0.2	169.0142
Protocatechuic acid	$C_7H_6O_4$	0.13	153.0184
3-Caffeoylquinic acid	$C_{16}H_{18}O_9$	1.03	353.0877
5-Caffeoylquinic acid	$C_{16}H_{18}O_9$	1.03	353.0877
Caffeic acid	$C_9H_8O_4$	0.2	179.0341
p-Coumaroyl quinic acid isomer	$C_{16}H_{18}O_8$	1.3	337.0931
p-Coumaroyl quinic acid isomer	$C_{16}H_{18}O_8$	1.3	337.0931
o/*m*-Coumaric acid	$C_9H_8O_3$	0.2	163.0391
Feruloylquinic acid	$C_{17}H_{20}O_9$	0.5	367.1035
p-Coumaric acid	$C_9H_8O_3$	0.1	163.0391
Quercetin-3-O-rutinoside (Rutin)	$C_{27}H_{30}O_{16}$	1.6	609.1466
Quercetin-3-O-galactoside (hyperoside)	$C_{21}H_{20}O_{12}$	1.33	463.0884
Quercetin-3-O-glucoside	$C_{21}H_{20}O_{12}$	1.33	463.0884
Kaempferol-O-hexoside	$C_{21}H_{20}O_{11}$	1.35	447.0935
Kaempferol-O-hexoside	$C_{21}H_{20}O_{11}$	1.35	447.0935
3,5-Dicaffeoylquinic acid	$C_{25}H_{24}O_{12}$	1.04	515.1196
3,4-Dicaffeoylquinic acid	$C_{25}H_{24}O_{12}$	1.04	515.1195
4-Caffeoylquinic acid	$C_{16}H_{18}O_9$	1.03	353.0877
1,4/4,5-Dicaffeoylquinic acid	$C_{25}H_{24}O_{12}$	1.04	515.1194
